# Why do psychiatric patients in Korea stay longer in hospital?

**DOI:** 10.1186/s13033-016-0110-6

**Published:** 2017-01-03

**Authors:** Agnus M. Kim

**Affiliations:** Department of Health Policy and Management, Seoul National University College of Medicine, 103 Daehak-ro, Jongno-gu, Seoul, 110-799 South Korea

**Keywords:** Psychiatric admission, Length of stay, Korea

## Abstract

Korea is the only developed country that saw an increase in the number of psychiatric beds with the longest average length of stay of psychiatric patients for the past decades. This phenomenon can be explained regarding the payment system, the law, and society. Korea is in a critical position concerning mental health policy. How it paves the way for reducing psychiatric admissions will provide a model for rearranging the interests of different social groups for the sake of a higher value, that of human rights.

## Background

Admissions of psychiatric patients have decreased in most developed countries [1]. With deinstitutionalization as a fundamental policy direction [2], many countries have implemented policies to reduce the number of beds and support outpatient- and community-based care [1].

In Korea, however, admission remains the foremost recourse in psychiatric treatment. In contrast with the general trend in most developed countries, the number of psychiatric beds in Korea has continually increased, and the length of stay of psychiatric patients in Korea has remained long for years [3]. While the number of psychiatric beds per 100,000 population decreased about 20% on average among the OECD countries over the past decades, Korea showed a 340% increase in psychiatric bed rate during the same period [4]. Additionally, the average length of stay of psychiatric patients in Korea was the longest among OECD countries at 116 days in 2011, which was about four times the average in OECD countries [5].

The management of psychiatric patients concerning psychiatric admission differs considerably among countries and this difference stems from each country’s different system and culture. However, given that many countries are pursuing a balance between community-based care and hospital-based treatment by decreasing the relative role of psychiatric admission, the high dependence on psychiatric admission observed in Korea may not be in the best interest of patients and needs to be addressed. The present commentary will investigate this issue with regard to the payment system, the law, and society.

## Payment system

The payment system affects the behaviours of health care providers, and the length of stay of psychiatric patients is also known to be influenced by the payment system [6]. The payment system is one of the main factors maintaining a high admission rate of psychiatric patients in Korea. Korea implements a compulsory National Health Insurance system, which covers 97% of the total population in Korea by providing benefits for disease, injury, childbirth, etc. [7]. The remaining 3% of the population are supported by the Medical Aid program for low-income people. While the insured should pay a certain portion of the health care costs and the co-payment varies according to the type of care (inpatient or outpatient) and level of institution [8], the government covers nearly all expenses for Medical Aid beneficiaries for medical services stated in the law.

Zero co-payment for Medical Aid beneficiaries can induce unnecessary inpatient admissions. Even given that possibility, however, the admission rate of Medical Aid psychiatric patients is inordinately high. While only 3% of the total population in Korea are Medical Aid beneficiaries, psychiatric admissions covered by Medical Aid account for 53% of the total psychiatric admissions and 64% of the total long-term psychiatric admissions in Korea [3]. It is difficult to explain this high proportion of Medical Aid beneficiaries in psychiatric admissions only as a corollary of the zero co-payment in admission costs, because the admission rate of Medical Aid beneficiaries from causes other than psychiatric disease is not as distinctively high as that of Medical Aid psychiatric patients.

Unlike usual Medical Aid reimbursement, whose extent varies depending on the amount of care received by the patient, the reimbursement for psychiatric treatment in Medical Aid is fixed as per diem. The gap between Medical Aid and National Health Insurance psychiatric patients in terms of average expense is more prominent in outpatient care. In the case of outpatient care, the reimbursement of Medical Aid psychiatric patients is fixed at about $2.4 a day, which is only one tenth of the average expense of National Health Insurance psychiatric patients.

**Fig. 1:**
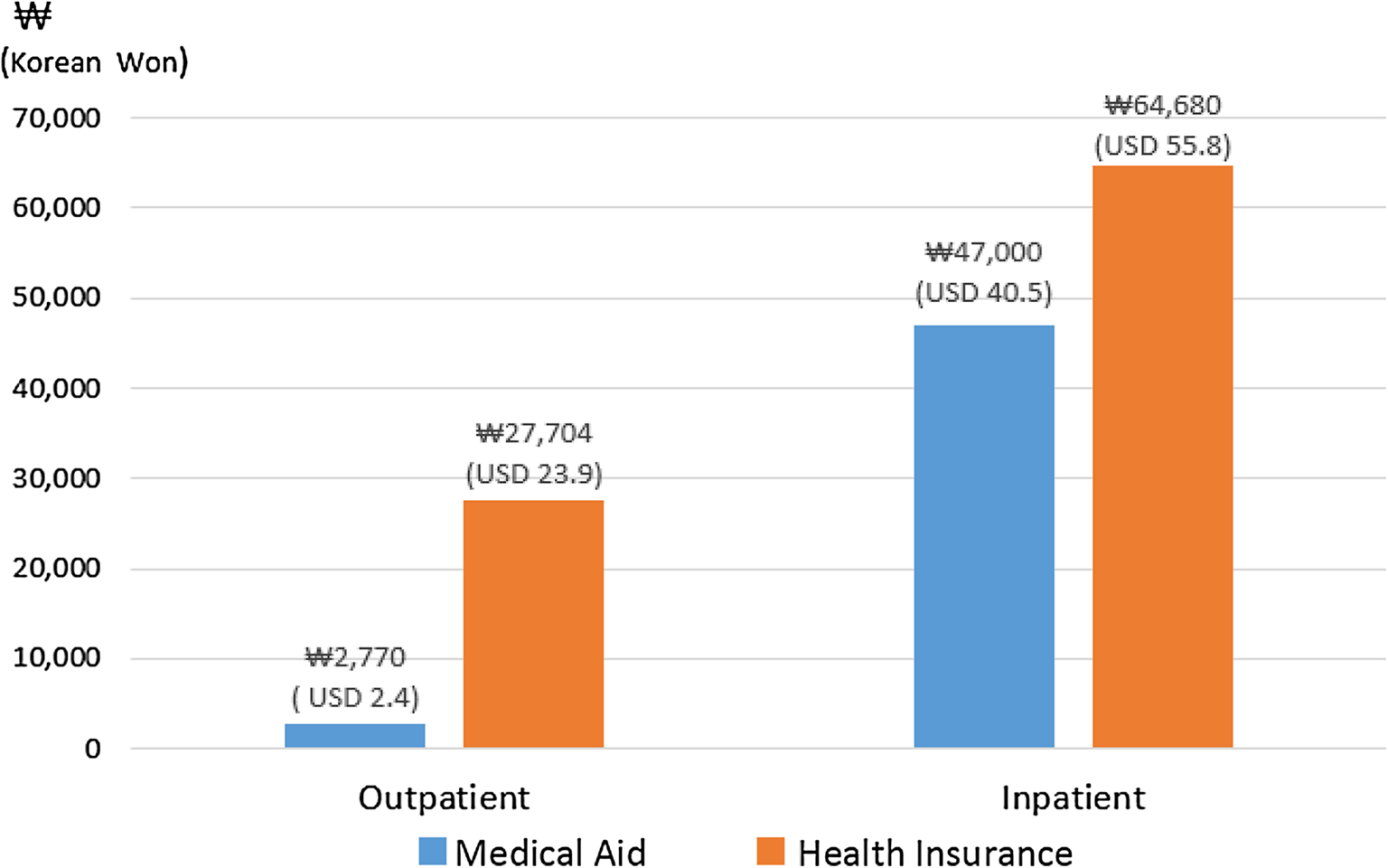
Reimbursement per day for the psychiatric patients in Korea according to insurance status. Reimbursement for National Health Insurance is an average value. Reimbursement for Medical Aid Inpatient is based on G2 (Reimbursement for Medical Aid Inpatient is differentiated according to the grade of the psychiatric hospital (G1–G5). This graph is based on the most common grade G2 which comprises about 68% of total psychiatric hospitals [9].) Data from the Ministry of Health and Welfare, Korea

The unduly low reimbursement for outpatient care of Medical Aid psychiatric patients limits treatment options in outpatient care for these patients. Regarding psychiatric inpatient care, however, the reimbursement in Medical Aid amounts to two-thirds of the average expense of a National Health Insurance beneficiary (Fig. 1). Considering the size of the gap in reimbursement, the quality of treatment for Medical Aid psychiatric patients is likely to be less compromised in inpatient than in outpatient care. Therefore, inpatient care becomes a better option than outpatient care for Medical Aid psychiatric patients.

Moreover, inpatient care offers the family of Medical Aid psychiatric patients an additional advantage; the family are relieved from the burden of caring for the patient through the hospitalization. Especially for families caring for severe chronic psychiatric patients, admission in itself can be an attractive choice, aside from the comparative advantage over outpatient care concerning reimbursement. Additionally, considering that the community-based care that should accompany outpatient care is not well established in Korea, long-term admission remains the least-worst choice for chronic psychiatric patients and their families. From the hospitals’ point of view, hospitalizing patients is a more attractive option not only because of the relatively better reimbursement rate for inpatient care but also because of the high possibility of a prolonged admission. Since hospitals can increase profit by minimizing expense under per diem reimbursement, they are motivated to induce and maintain hospitalizations. As a result, Medical Aid psychiatric outpatients are induced to switch to inpatient care, and those already hospitalized remain as inpatients. In 2014, the average length of stay of Medical Aid psychiatric patients was 228 days and half of them were admitted for more than 361 days [9].

## Law—involuntary admission

Another important factor that facilitates psychiatric admission is the Mental Health Act, which regulates involuntary psychiatric admission. Involuntary psychiatric admissions account for about 80% of all psychiatric admissions in Korea [4], which is noticeably higher when compared with other developed countries. Involuntary psychiatric admission has a legal basis on the Mental Health Act.

The Mental Health Act (paragraph 1 of Article XXIV) states that the director of a mental institution may hospitalize a mentally ill person in cases where a psychiatrist judges that hospitalization is necessary, with the consent of two persons responsible for providing protection to the mentally ill person. Although requiring the judgement of a psychiatrist for the involuntary psychiatric admission, the Act specifies no explicit legal or medical grounds for the judgement of the psychiatrist. Therefore, the Act involves the risk of being abused for involuntary admissions that are not medically justified.

Although limiting the length of admission to six months, the Act actually facilitates long-term hospitalization. The Act (paragraph 3 of Article XXIV) states that the director of the mental institution shall request the head of administration to examine a hospitalization every six months when a psychiatrist has stated that the continued hospitalization is necessary, with the consent of a person responsible for providing protection to the patient. However, in practice, the consent of a person responsible for providing protection can play a decisive role in prolonging admission. This is especially true for chronic psychiatric patients, who generally do not show marked improvement and are therefore not likely to offer a definite basis for changing the psychiatrist’s previous decision on involuntary admission.

The Act does not only promote the lengthening of admission but also can be abused as a means to admit a patient who is not eligible for admission. According to Article XXIV of the Act, it is not impossible to compulsorily hospitalize a person who is not so severely ill as to be admitted, if two persons responsible for providing protection agree to the admission and a psychiatrist judges it necessary. In practice, however, involuntary admissions which are medically unjustifiable have been frequently reported in Korea. Although Article XXVIII of the Act states that the Basic Mental Health Deliberative Committee examines whether to continue an admission or treatment to which an objection has been raised, the committee scarcely functions [10, 11]. In 2008, only 4.8% of the patients who raised claims for discharge orders actually obtained discharge orders [10]. Even if ordered to be discharged, more than half of the patients were admitted again, and half of those readmitted were re-hospitalized within a day after discharge [10].

In 2014, a 60-year-old Korean woman filed a constitutional review with the Constitutional Court of Korea claiming that Article XXIV of the Mental Health Act encroaches personal freedom. This woman, who had only suffered from mild involutional depression, was hospitalized compulsorily with the consent of her two children and the decision of a psychiatrist. She insisted that she had suffered no serious mental illness that could lead her to threaten her own or others’ safety or that required hospitalization. On Sept. 29, 2016, the Constitutional Court of Korea decided that Article XXIV of the Mental Health Act was incompatible with the Constitution [11]. In the decision, the Constitutional Court of Korea stated that (1) the Act does not provide the explicit criteria for involuntary admission and can therefore be abused by persons responsible for providing protection and hospitals, (2) the examination of the legitimacy concerning involuntary admission or its extension is not being properly performed, and (3) the Act can infringe personal freedom due to excessive restriction on the freedom of the psychiatric patient [11].

## Society—admission for whom?

Despite a steady increase in the number of psychiatric beds in Korea in the past decades, the circumstances surrounding hospitalized patients have not improved accordingly. According to a report from the National Human Rights Commission of Korea, 45% of psychiatric inpatients have experienced seclusion or restraints, 9.3% have experienced violence from hospital employees, and 10.5% violence from other inpatients [12]. From 2001 to 2012, the number of psychiatric beds in private psychiatric hospitals doubled, while those in public psychiatric hospitals remained almost the same, and there was about 20% increase in general hospitals [13]. Considering that private hospitals would be more willing to seek profit than public hospitals, and that treatment in private psychiatric hospitals is chiefly based on hospitalization, it is difficult to deny that the profit motive of private capital underlies the increase in psychiatric beds. Poor conditions in some private psychiatric institutions, such as high numbers of patients per psychiatrist, poorly equipped hospital facilities, and violent treatment or restraint abuse to control a high number of patients with less personnel [14], prove that hospitals continue seeking profit in inappropriate ways even after hospitalizing patients.

The increase in the number of psychiatric beds and sacrifice in the quality of psychiatric treatment can be attributed, to a large extent, to the government policy. It would have been impossible to establish and maintain psychiatric hospitals without the permission and support of the government. In addition, patients’ families who acquiesce to hospitalizing patients under circumstances where no better alternatives are available are not free from responsibility for this situation. However, society, which has condoned this situation for more than decades, is the most accountable. If society were aware of the problem and continued to raise it as an issue, more psychiatric patients in Korea could lead better lives outside the hospital. The status quo of psychiatric admission in Korea is based on the tacit agreement of members of society.

## Conclusion

Although low reimbursement for Medical Aid psychiatric patients and legal problems regarding involuntary admission have been pointed out, the reimbursement rate for Medical Aid patients has been frozen for eight years and no significant modification has been made to the Mental Health Act concerning involuntary psychiatric admission since its introduction in 1995. The recent decision about the unconstitutionality of Article XXIV of the Mental Health Act is encouraging. However, in order to revise the law, the government should resolve differences among various social entities including patients, patients’ families, psychiatrists, hospitals, and the general public, which would be an onerous task for everyone.

Korea is in a critical position regarding mental health policy in that it is the only developed country that goes against the trend of deinstitutionalization and perhaps also against the best interest of psychiatric patients. How it paves the way for reducing psychiatric admissions will provide a model for rearranging the interests of different social groups for the sake of a higher value, that of human rights. Korea is expected to present an example for the rest of the world.

## Abbreviation

OECD: Organisation for Economic Co-operation and Development
